# Tracing weak neuron fibers

**DOI:** 10.1093/bioinformatics/btac816

**Published:** 2022-12-26

**Authors:** Yufeng Liu, Ye Zhong, Xuan Zhao, Lijuan Liu, Liya Ding, Hanchuan Peng

**Affiliations:** SEU-ALLEN Joint Center, Institute for Brain and Intelligence, Southeast University, Nanjing, Jiangsu 210096, China; SEU-ALLEN Joint Center, Institute for Brain and Intelligence, Southeast University, Nanjing, Jiangsu 210096, China; SEU-ALLEN Joint Center, Institute for Brain and Intelligence, Southeast University, Nanjing, Jiangsu 210096, China; SEU-ALLEN Joint Center, Institute for Brain and Intelligence, Southeast University, Nanjing, Jiangsu 210096, China; SEU-ALLEN Joint Center, Institute for Brain and Intelligence, Southeast University, Nanjing, Jiangsu 210096, China; SEU-ALLEN Joint Center, Institute for Brain and Intelligence, Southeast University, Nanjing, Jiangsu 210096, China

## Abstract

**Motivation:**

Precise reconstruction of neuronal arbors is important for circuitry mapping. Many auto-tracing algorithms have been developed toward full reconstruction. However, it is still challenging to trace the weak signals of neurite fibers that often correspond to axons.

**Results:**

We proposed a method, named the NeuMiner, for tracing weak fibers by combining two strategies: an online sample mining strategy and a modified gamma transformation. NeuMiner improved the recall of weak signals (voxel values <20) by a large margin, from 5.1 to 27.8%. This is prominent for axons, which increased by 6.4 times, compared to 2.0 times for dendrites. Both strategies were shown to be beneficial for weak fiber recognition, and they reduced the average axonal spatial distances to gold standards by 46 and 13%, respectively. The improvement was observed on two prevalent automatic tracing algorithms and can be applied to any other tracers and image types.

**Availability and implementation:**

Source codes of NeuMiner are freely available on GitHub (https://github.com/crazylyf/neuronet/tree/semantic_fnm). Image visualization, preprocessing and tracing are conducted on the Vaa3D platform, which is accessible at the Vaa3D GitHub repository (https://github.com/Vaa3D). All training and testing images are cropped from high-resolution fMOST mouse brains downloaded from the Brain Image Library (https://www.brainimagelibrary.org/), and the corresponding gold standards are available at https://doi.brainimagelibrary.org/doi/10.35077/g.25.

**Supplementary information:**

Supplementary data are available at *Bioinformatics* online.

## 1 Introduction

Understanding the morphology and connectivity of neurons is crucial for cell typing, wiring characterization and simulation. Amount of neuronal reconstructions have been collected and archived on open-sharing websites such as NeuroMorpho.org (Ascoli *et al.*, 2007), FlyCircuits (Chiang *et al.*, 2011) and FlyLight (Jenett *et al.*, 2012), and Initiatives including DIADEM challenge (Brown *et al.*, 2011) and BigNeuron project (Peng *et al.*, 2015). However, the production of high-quality single-neuron reconstructions in high throughput is still challenging.

A promising way to fill the gap between primary data (i.e. microscopic images) and secondary data (i.e. morphological reconstructions) is automatic neuron tracing. To address this problem, researchers developed numerous automatic or semi-automatic algorithms (Al-Kofahi *et al.*, 2002; Chen *et al.*, 2015; Coleman *et al.*, 1977; Garvey *et al.*, 1973; Glaser and Glaser, 1990; Liu *et al.*, 2016, 2017; Peng *et al.*, 2011, 2017; Quan *et al.*, 2016; Xiao and Peng, 2013; Yang *et al.*, 2019; Zhou *et al.*, 2016, 2021), as well as many pre-tracing and post-tracing algorithms, including image enhancement (Liang *et al.*, 2017), segmentation (Huang *et al.*, 2020; Li and Shen, 2020; Li *et al.*, 2017b; Liu *et al.*, 2018; Wang *et al.*, 2019, 2021; Zhou *et al.*, 2018), post-processing (Li *et al.*, 2017b, 2019c), critical points detection (Chen *et al.*, 2021; Liu *et al.*, 2019; Shen *et al.*, 2021; Tan *et al.*, 2020) and ensembling (Wang *et al.*, 2017). Nevertheless, most of these algorithms are designed for well-selected images, and the tracing quality for whole brain light microscopic images could not meet the requirement for morphological and physiological analyses (Manubens-Gil *et al.*, 2022).

Many obstacles make neuron reconstruction challenging (Basu *et al.*, 2013). Although current microscopes, e.g. fluorescence micro-optical sectioning tomography (fMOST) (Gong *et al.*, 2016), generate whole-brain images in submicron resolution with high quality, considerable fibers are often weakly imaged (Li *et al.*, 2019b). These weak fibers are difficult to identify when surrounded by noisy backgrounds.

**Fig. 1.**
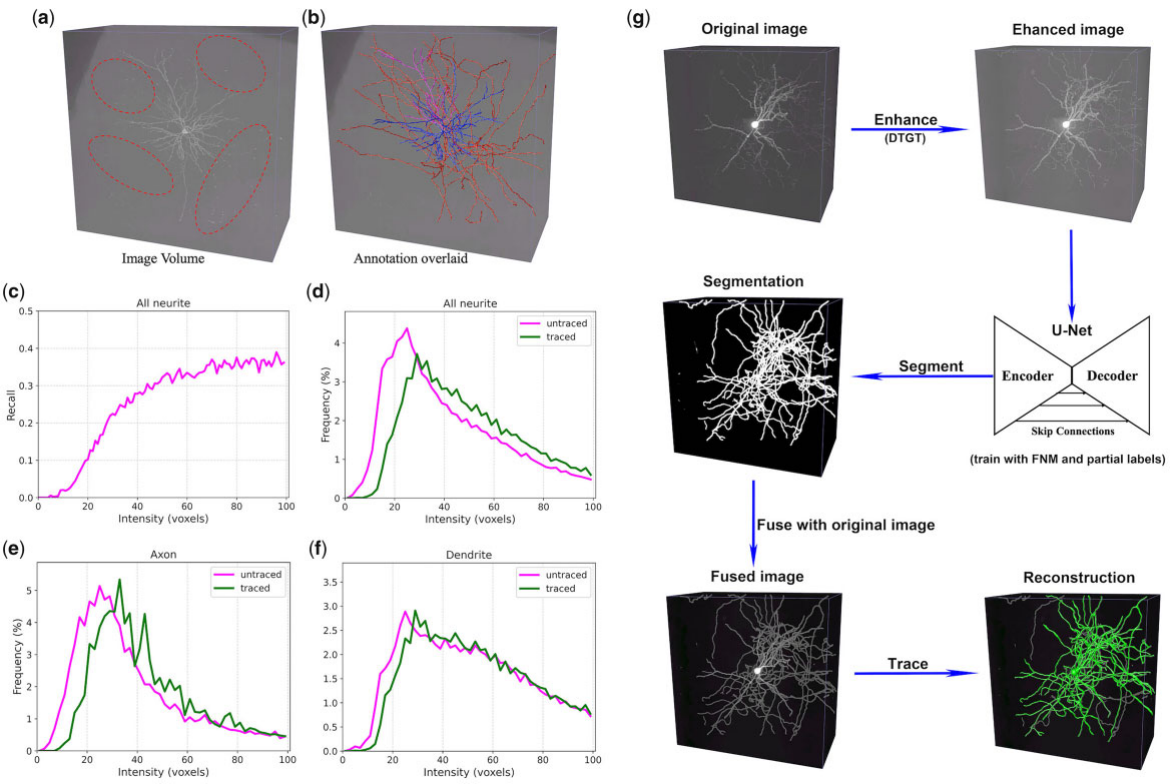
Schematic illustration of NeuMiner. (**a**) An example of weak fibers (fibers within the ellipses) consisted of low-intensity voxels. (**b**) Gold standard swc overlaid on the image. (**c**) Relation between voxel intensity and recall of APP2 reconstructions. (**d–f**) Intensity distributions between traced and untraced neurites, for all neurites, axons and dendrites. The weak fibers (voxel intensity < 20) are not well recognized, and untraced fibers have significantly weaker fibers. (**g**) Workflow of NeuMiner tracing. The original image is firstly enhanced with a modified gamma transformation (DTGT), and subsequently segmented with a U-Net. The segmentation network is trained with FNM to improve the weak fiber recognition. Training with partial labels can identify most fibers. The segmentation is then fused with the original image and traced using existing tracing algorithms, like APP2, to get the final reconstruction

In this article, we proposed a neuron tracing module called NeuMiner with a focus on tracing weak fibers (Fig. 1). To this end, we introduced a false negative mining (FNM) strategy into the segmentation network by adjusting the losses of false-negative voxels in training. We also applied a modified gamma transformation, derivative truncated gamma transformation (DTGT), to the input images by truncating the derivative of the gamma function to no <1. We also demonstrated neuron segmentation with partially annotated images can identify 93% more signals on average. Overall, NeuMiner empowers base tracers with much higher recall for weak fibers, based on extensive evaluations of the newly released single-neuron dataset.

## 2 Materials and methods

### 2.1 Dataset

The recently released brain-wide single-neuron dataset is adopted in this article (Peng *et al.*, 2021). The dataset contains 53 high-quality imaged brains and over 1700 neurons manually annotated from 34 brains by experts. We downloaded 1726 neurons from 31 brains (three brains are failed to download. ID: 191807, 182724 and 210254). Resolutions of images vary from (0.2 µm × 0.2 µm × 1.0 µm) to (0.35 µm × 0.35 µm × 1.0 µm). Every neuron is cropped to constant size (1024 × 1024 × 512) centered at the soma position and then down-sampled to (512 × 512 × 256). The crop size is chosen so that it contains the majority of dendrites and considerable axons while keeping the computation workload affordable. 1676 neurons from 29 brains are randomly split into train, validation and test set, containing 1357, 168 and 151 neurons, respectively. The other 50 neurons from 2 new independent brains are used for validation of unseen data. Images with more than one somata are removed in validation and test sets to get rid of densely packed neurons, which is out of the scope of this article. Therefore, 61 and 59 samples are used for validation and testing, and 29 neurons are used for benchmarking of new brains. The validation set is used for parameter searching, and the final results are evaluated on the test set. Image resolutions and sizes are in (x, y, z) order unless explicitly stated.

### 2.2 Segmentation network

A vanilla 3D UNet (Ronneberger, 2017) is utilized. The model is trained with randomly cropped (160 × 160 × 128) volumes. Diverse augmentations, including cropping, gamma transformation, Gaussian noises, flipping and resizing, are randomly sampled for each input image.

Instance normalization (Ulyanov *et al.*, 2017) is applied before every nonlinear activation layer. Networks are trained with Nesterov-based SGD, with an initial learning rate of 0.01, weight decay of 3e−5, and momentum of 0.99. Dice loss (Milletari *et al.*, 2016) and cross-entropy loss are applied to the last layers of the last two blocks. All models are trained for 15 200 iterations using Pytorch (version 1.8) with Automatic Mixed Precision acceleration enabled.

### 2.3 Label generation

Segmentation labels are generated according to manual annotated gold standards (i.e. *swc* files), by resampling annotated nodes to connective points in 3D space. Each point is represented by a uniform width of (3 × 3 × 1) voxels. The somata are overlaid with a fixed size (18 × 18 × 6) cuboid for both input images and label images. Ellipsoids with different radii are also evaluated on the validation set, they share similar performances. Only part of the neurons is annotated, and there may be considerable numbers of unlabeled fibers in each image volume.

### 2.4 False negative mining

Segmentation is a per-pixel/voxel classification problem (Zhou *et al.*, 2018) and is usually trained with pixel/voxel-wise cross-entropy loss or dice loss. Although the foreground in both input image and label image shows co-occurrence, their relationship is not guaranteed while inference. Moreover, the fibers are very diverse in intensity, many of them are discontinuous and weak (fibers in ellipses in Fig. 1a).

Those weak fibers are challenging for a segmentation network, resulting in false-negative classification. A FNM strategy is introduced to alleviate the problem. The detailed implementation of it is elaborated as follows.

For per-voxel loss, it can be formulated as

$$L=\frac{1}{N}\sum_{i=1}^{B}\sum_{j=1}^{C}\sum_{k=1}^{Z}\sum_{l=1}^{Y}\sum_{m=1}^{X}w_{i,\;j,\;k,\;l,\;m}\cdot L_{i,\;j,\;k,\;l,\;m}$$

where B, C, Z, Y, X are the batch size, channel number, image shape in z, y, x-axes, respectively. N is the number of voxels calculated as B·C·Z·Y·X. $w_{i,\;j,\;k,\;l,\;m}$ and $L_{i,\;j,\;k,\;l,\;m}$ are weight and loss for the voxel (i, j, k, l, m). The loss is universal to all per-voxel losses.

Firstly, the on-the-fly false-negative sample set FN is extracted via

$$\mathrm{FN}=F\cap \left(P<0.5\right),$$

where F is the foreground voxel set generated according to the labels, and P is the segmentation.

Once FN is estimated, the loss weights for the false-negative voxels can be adjusted as

wi, j, k, l, m=wFN, pi, j, k, l, m∈FN1.0, pi, j, k, l, m∉FN

In this article, false-negative weight $w_{\mathrm{FN}}$ is set as 1.5 by line search. Notably, only false-negative weights are increased, as there are considerable numbers of unlabeled fibers.

### 2.5 Derivative truncated gamma transformation

Gamma transformation is a nonlinear image transformation that follows gamma function (i.e. power function) expressed as

$$g\left(x\right)=x^{\gamma },\;x\in [0,1]$$

where image voxel x are normalized in range [0,1] in prior. To amplify the weak fibers, γ should be smaller than 1.0.

**Fig. 2.**
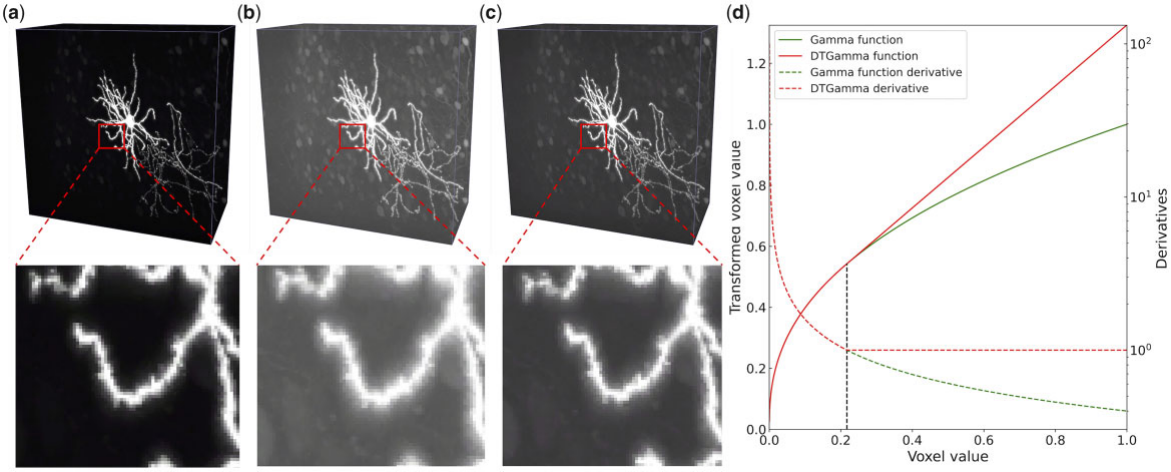
Comparison of gamma transformation and DTGT. (**a**) Original image. (**b**) Gamma transformation of the original image. (**c**) DTGT of the original image. (**d**) Illustration of gamma and derivative truncated gamma function, with γ = 0.4 for both functions. The high-intensity fibers highlighted by rectangles exemplify the halo effect after gamma transformation. These regions are zoomed in for better visualization. While both transformations enhance the weak fibers significantly, the proposed DTGT suppresses the diffusive halo artifact around the bright fibers

One drawback of the gamma function is that its derivatives of higher intensity region (voxel value x near 1.0) are smaller than 1 (Fig. 2d), which can be inferred from the derivative function

$$g^{\prime }\left(x\right)=\gamma \cdot x^{\gamma -1},\;x\in [0,1]$$

Therefore, the contrast of brighter fibers will be attenuated after applying the gamma function. This may cause a diffusive effect which we call the halo effect, as illustrated in Figure 2b.

We propose to replace the higher intensity region of the gamma function with the identity function so that the derivatives are no <1. The new function is formulated as

g^x=xγ, x∈[0,δ]x-δ+δγ, x∈[δ,1]

where δ is truncation point where the derivative equals 1, calculated as

$$\delta =e^{\frac{\ln\gamma }{1-\gamma }}$$

Voxel values are standardized after the transformation to keep the range unchanged.

We call the new function $\hat{g}\left(x\right)$ derivative truncated gamma function, and transformation based on it as DTGT. The truncated function is illustrated in Figure 2d. As expected, the halo effect is restrained as shown in Figure 2c.

### 2.6 Tracing

Segmentation confidences are fused with the original image through a simple linear combination

$$\hat{x_{i}}=\alpha \cdot x_{i}+\left(1-\alpha \right)\cdot p_{i},$$

where $x_{i}$ and $p_{i}$ are corresponding values on input image and segmentation, and the value of α is 0.8, which is the same as Li *et al.* (2017a).

The fused image is subsequently traced with existing tracers. In this article, APP2 (Xiao and Peng, 2013) and SmartTracing (Chen *et al.*, 2015) are adopted to evaluate our methods, similar to Li *et al.* (2017a). APP2 is a faster and more accurate variant of APP1 (Peng *et al.*, 2011), and it is still one of the most robust and popular methods ever since its release. By integrating SVM-based segmentation into the APP2 baseline, SmartTracing is a prevalent alternative. Another critical reason for choosing these methods is that these two methods are relatively tuning-free, and tracing with default parameters will get sufficient good results.

## 3 Results

### 3.1 NeuMiner improves the segmentation of weak fibers

The recall of weak fibers (5.1%) is much lower than other fibers (∼36%, Fig. 1c) and they are more likely to be untraced, especially for axons (Fig. 1d–f). Global contrast normalization, e.g. histogram equalization, improves the contrast at the expense of over-exposure (Supplementary Fig. S1). Adaptive enhancement, e.g. contrast limited adaptive histogram equalization (CLAHE), overcomes this problem and successfully enhances the weak fibers (Supplementary Fig. S1). Nevertheless, the patch-based contrast normalization CLAHE is highly correlated to nearby signals. If the fibers are close to high-intensity voxels, they will be suppressed after transformation (yellow arrows in Supplementary Fig. S1). Our proposed DTGT enhances the weak fibers comparably to CLAHE, and the enhancement is independent of nearby signals (Supplementary Fig. S1).

**Fig. 3.**
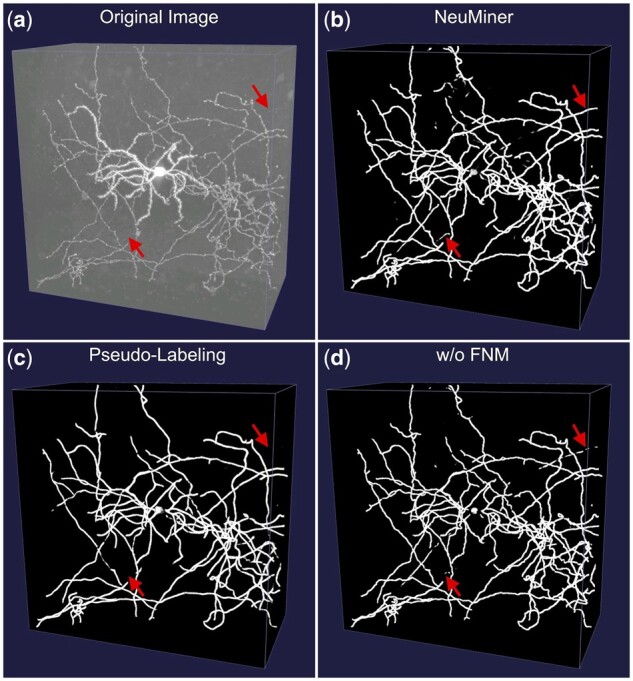
Segmentation results under different conditions. (**a**) An exemplar image volume from brain 18455. (**b**) Corresponding segmentation generated by NeuMiner. (**c**) Segmentation generated by pseudo-labelling experiment, whose label for segmentation network is generated from APP2. (**d**) Segmentation without FNM applied. While most fibers are well segmented for all methods, weak fibers pointed out by the arrows are better recognized by NeuMiner

The FNM strategy is critical for the higher recall of weak fibers. Theoretically, FNM increases the recall of all fibers indiscriminately. Since the strong fibers (fibers of high intensity) are easily classified, the weak fibers benefit preferentially. As expected, the strong fibers are comparably recognized regardless of whether FNM is used, while the very weak fibers are better segmented when FNM is enabled (Fig. 3b).

**Fig. 4.**
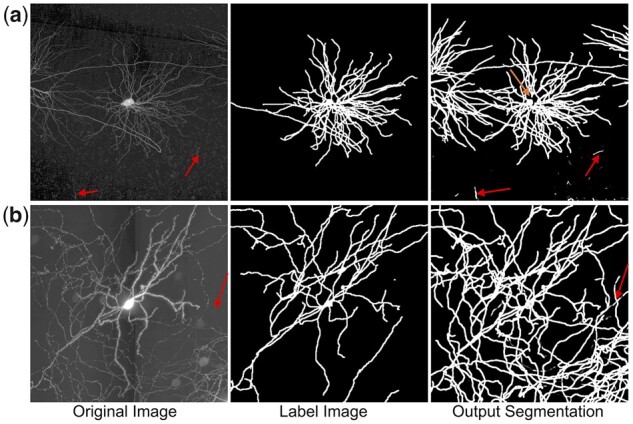
Examples of segmentation with partial labels. (**a, b**) are examples of two different neurons. The first columns are original image volumes, the second and last columns are label images and segmentations. The arrows highlight the very weak fibers that identified by our method, and the topmost arrow in the top-right penal points out the disconnected prediction near soma. Despite large numbers of fibers are not annotated, NeuMiner correctly recognized most unlabeled fibers, as well as many very weak fibers

NeuMiner successfully recognized most fibers, including unlabeled ones (Fig. 4) and very weak fibers (Fig. 4, pointed out by arrows). The average ratio of foreground voxel number in segmentation to that of label images is 1.93 ± 0.69, i.e. 93% more voxels are identified. Meanwhile, both discrete Gaussian Noises (Fig. 4a) and oval-shaped plaques (Fig. 4b) are suppressed. Soma body is occasionally disconnected from other fibers (Fig. 4). This is caused by the lack of soma segmentation labels, and the artificial uniform labeling is ambiguous for determining the highly diverse soma bodies. The disconnection can be rescued by fusing with the original image.

Except for training with partial labels adopted in this article, weakly supervised learning methods that leverage automatic tracing reconstructions as pseudo-labels are proved to be effective in many large-scale datasets (Huang *et al.*, 2020). For comparison, we applied automatic tracer APP2 to all neurons in the train set and used the reconstructions as training labels. It is comparable for the majority of fibers, but most weak fibers are missed (Fig. 3c). Statistical analysis on the tracing results confirmed the poor recognition on axons (average ${\mathrm{SD}}_{12}$ 12.53, compared to our method 1.87).

We also tested a recently proposed neuronal image-enhancing method, imPreProcess (Guo *et al.*, 2022), which was demonstrated to enhance signal-background contrast and improve reconstruction quality by a large margin. Results on our dataset show it optimized signal recognition considerably (average ${\mathrm{SD}}_{12}$ is 6.11), but is inferior to our method in weak fiber recognition.

### 3.2 NeuMiner improves the tracing of weak fibers

**Fig. 5.**
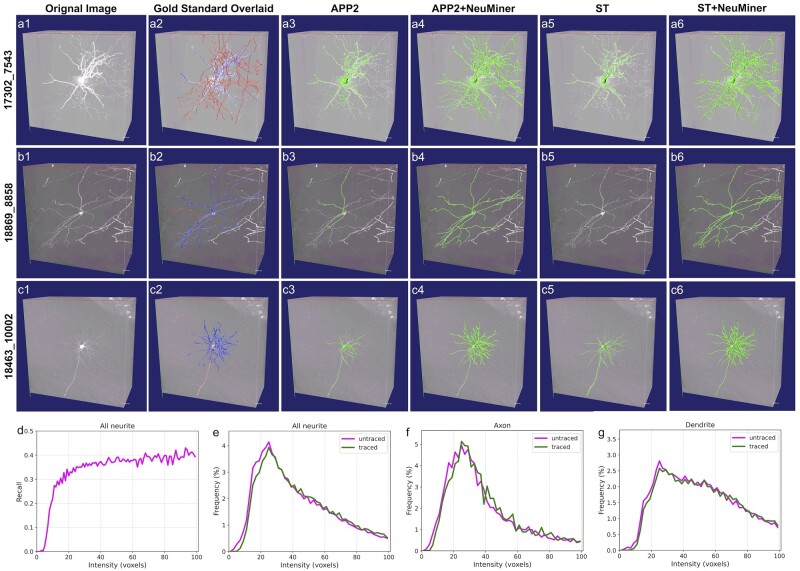
Tracing improvement of NeuMiner. (**a1–c1**) Neuron image volumes from different brains (brain id: 17302, 18869 and 18463). (**a2–c2**) Neuron image volumes with gold standards overlaid. (**a3–c3, a4–c4, a5–c5** and **a6–c6**) Corresponding reconstructions by APP2, APP2 with NeuMiner enabled, SmartTracing (ST) and ST with NeuMiner enabled. Considerable weak fibers are missed by both APP2 and ST, while most of them are identified when NeuMiner is enabled. (**d**) Relation between voxel intensity and recall of NeuMiner enhanced APP2 reconstructions. (**e–g**) Intensity distributions between traced and untraced neurites, for all neurites, axons and dendrites. The recall of weak fibers (27.8%) is significantly improved, compared to vanilla APP2 reconstruction (5.1%) as shown in Figure 1. The intensity distributions for untraced and traced fibers are similar when NeuMiner is applied

While both APP2 and SmartTracing perform well on most neurons, they are not good at tracing weak fibers by design (Fig. 1c). Weak fibers are frequently missed by both APP2 and SmartTracing. It is not only observed on axon tracing (Fig. 5a3 and a5) but also on dendrites (Fig. 5b3, b5, c3 and c5). The problem is alleviated when combined with NeuMiner, and most fibers are successfully identified (Fig. 5a4, a6, b4, b6, c4 and c6).

Quantitative analysis is conducted for a more comprehensive evaluation. We first evaluated with three mostly cited metrics: spatial distance (SD), substantial spatial distance (SSD) and percentage of different structure (PDS) (Peng *et al.*, 2011) on the test set. SD is the average of minimal distances of points in the comparing neuron for all voxels in the estimating neuron, SSD is a modified SD metric that takes into consideration only distances larger than two voxels, and PDS, which is defined as minimal reciprocal SD larger than two voxels. These metrics are asymmetric, thus they are represented by label-to-prediction ($B_{12}$), prediction-to-label ($B_{21}$), and the average of these two (B), where B represents any kind of above metrics (SD, SSD, PDS). $B_{12}$ is highly correlated to recall, and $B_{21}$ to precision. Lower is better.

All metrics show consistent conclusions. The recall of NeuMiner-enabled versions is significantly higher than vanilla APP2 and SmartTracing, with ${\mathrm{SD}}_{12}$ distances decreased by 87.6 and 95.6%, respectively (Table 1). Similar advantages are also observed on ${\mathrm{SSD}}_{12}$ and ${\mathrm{PDS}}_{12}$. On the other hand, the precision ($B_{21}$) is comparable. Specifically, all $B_{21}$ distances are marginally increased compared to APP2, while NeuMiner has a minor advantage to SmartTracing baseline as two out of three metrics are smaller. Overall, the averaged distances of NeuMiner are much better, with 49.2%, 25.7% and 42.3% distances reduction for APP2, and 62.6%, 36.2% and 51.2% for SmartTracing.

**Table 1.** Quantitative comparison between base tracers and corresponding NeuMiner enabled versions

	${\mathrm{SD}}_{12}/{\mathrm{SD}}_{21}/\mathrm{SD}↓$			${\mathrm{SSD}}_{12}/S{\mathrm{SD}}_{21}/\mathrm{SSD}↓$	${\mathrm{PDS}}_{12}/{\mathrm{PDS}}_{21}/\mathrm{PDS}↓$	Topology-based metrics↑		
	All neurites	Dendrite^a^	Axon			OPT-J	OPT-P	OPT-G
APP2	11.82/**5.47**/8.64	4.42/-/-	17.86/-/-	23.04/**15.67**/19.35	0.36/**0.15**/0.26	0.80 ± 0.12	0.69 ± 0.17	0.65 ± 0.23
+NeuMiner	**1.47**/7.31/**4.39**	**0.93**/-/-	**1.87**/-/-	**6.36**/22.40/**14.38**	**0.10**/0.20/**0.15**	**0.88 ± 0.07**	**0.82 ± 0.11**	**0.80 ± 0.15**
ST^b^	23.93/16.60/20.26	18.39/-/-	29.12/-/-	31.54/**27.30**/29.42	0.46/0.35/0.41	0.75 ± 0.16	0.62 ± 0.19	0.55 ± 0.25
+NeuMiner	**1.06/14.08/7.57**	**0.78**/-/-	**1.24**/-/-	**5.49**/32.08/**18.78**	**0.09/0.31/0.20**	**0.82 ± 0.10**	**0.76 ± 0.13**	**0.73 ± 0.18**

*Note*: The best values in each category are highlighted in bold. Down arrows indicate that the smaller the metrics, the better the results. Up arrows are the opposite.

a For distance metrics of separate axon or dendrite, $B_{21}$ and B is meaningless as a considerable proportion of neurites are removed, so they are represented as -/-.

b Forty-three reconstructions are used for evaluation of SmartTracing (ST) based methods, as the others are not successfully reconstructed by ST.

The much lower $B_{12}$ distances indicate higher weak fiber tracing of NeuMiner. This is verified by the smaller average voxel intensity of extra-traced fibers (0.79 ± 0.68, ratio to the average intensity of all APP2 traced voxels). Visual inspection confirms this assumption. Large numbers of weak fibers are recognized by NeuMiner, but not by APP2 and SmartTracing (Fig. 5). The improvement varies for different neurite types: the average distance ${\mathrm{SD}}_{12}$ of the axons is decreased by 89.5% (from 17.86 to 1.87, Table 1), compared to 79.0% (from 4.42 to 0.93, Table. 1) for dendrites. Overall, the recall of weak fibers is increased from 5.1 to 27.8% (Figs 1c and 5d). The improvement is more prominent for axons, which increased by 6.4 times, compared to 2.0 times for dendrites. The average ${\mathrm{SD}}_{12}$ distances for axons are 1.87 and 1.24 voxels, which are even smaller than dendrite distances of the original APP2 and SmartTracing (Table 1).

A straightforward alternative to increase the recall of weak fibers is tracing with a lower background threshold. To evaluate the solution, we decreased the background threshold by half of the standard deviation of the input image, and re-run APP2. The average ${\mathrm{SD}}_{12}$ distance decreased (from 11.82 to 5.48), while the average ${\mathrm{SD}}_{12}$ distance increased dramatically (from 5.47 to 19.00), resulting in an increased average distance discrepancy (${\mathrm{SD}}_{12}$, from 8.64 to 12.24). Therefore, a smaller background threshold does not solve the tracing of weak fibers.

For a comprehensive assessment, three topology-based metrics, junction-based metric OPT-J path-based metric OPT-P and subgraph-based metric OPT-G (Citraro *et al.*, 2020) are also calculated. By fixing the weaknesses of existing metrics, the above three metrics are demonstrated to be superior on road segmentation. When combined with NeuMiner, both APP2 and SmartTracing show significant improvement for all those three metrics, especially for OPT-P and OPT-G (Table 1).

We also applied our method to another 26 neurons from two new brains, and the improvements of NeuMiner are again confirmed based on the performance on most metrics (Supplementary Table S2).

### 3.3 Ablation study

Ablation studies were carried out to reveal the contribution of each part. Since all three metrics share similar patterns (Table 1), this time we only use SD for simplicity. Quantitative analyses for these studies are summarized in Supplementary Table S1. The FNM strategy is the principal contributor among all tricks. When it is removed, some weak fibers are not well identified (Fig. 3d), and the overall ${\mathrm{SD}}_{12}$ distance increases from 1.47 to 2.67. The deterioration is especially profound for axon, (increases from 1.87 to 3.45), highlighting the necessity of the FNM strategy for achieving a high fiber recognition.

The DTGT also improves weak fiber recognition. The overall ${\mathrm{SD}}_{12}$ is also lower than baseline. Note that the increase of ${\mathrm{SD}}_{12}$ is mainly on the axons, which increased by 0.29 voxels from 1.87 to 2.16, while the dendrite distance keeps unchanged. This is reasonable as DTGT has a larger slope at a small value region, and only enhances the contrast for weak fibers.

Without truncation, i.e. vanilla gamma transformation, the recall of both dendrite and axon keeps unchanged, but the precision is slightly worse. Finally, if the image is not fused with segmentation, the ${\mathrm{SD}}_{12}$ distances for dendrite, axon, and all neurites are similar to our method, but the ${\mathrm{SD}}_{12}$ distance is 12 times larger than our baseline, highlighting the great noise discrimination power of the segmentation network, consistent with previous studies. When all those tricks are turned off, i.e. the original APP2, the performance is much worse as previously discussed.

### 3.4 Single-neuron tracing

Single-neuron tracing is highly challenging for long-projection neurons across multiple brain regions. We tested the performance of NeuMiner on single-neuron tracing, using UltraTracer (Peng *et al.*, 2017) plugin in Vaa3D (Peng *et al.*, 2010).

Results show NeuMiner improves the single-neuron tracing on most metrics and categories. The average distances (the last values of *All neurites* item in Supplementary Table S3) of the NeuMiner-enabled version for all neurites are lower, and it is more prominent on axon recognition. While reconstructions of UltraTracer are significantly shorter than gold standards (path length ratio to gold standards is 0.348 ± 0.279), NeuMiner enabled UltraTracer to get reconstructions with path length similar to gold standards (path length ratio to gold standards is 0.804 ± 0.823).

**Fig. 6.**
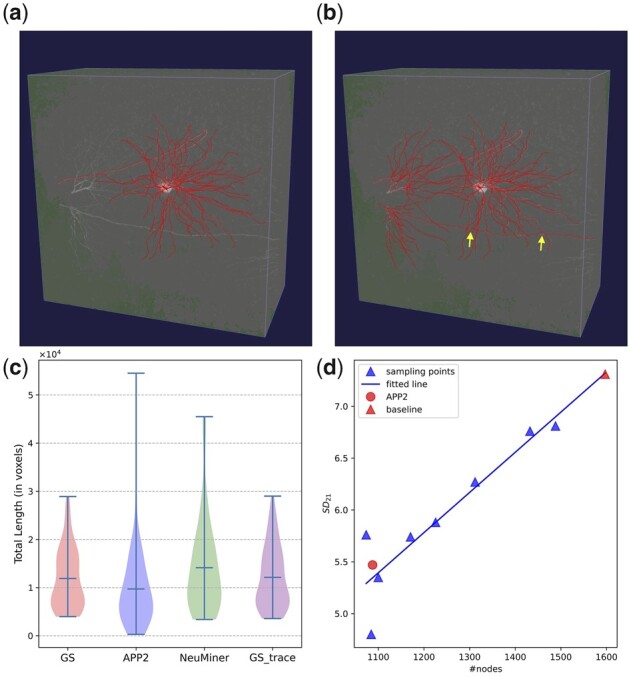
Fiber crossing and over-tracing. (**a**) Image volume with gold standard overlaid. (**b**) Image volume with reconstruction overlaid. Inter-crossing is pointed out by arrows. (**c**) Violin plot for distributions of total path length. GS, APP2, NeuMiner and GS_trace are total path length distributions for gold standards, APP2 reconstructions, reconstructions of APP2 with NeuMiner enabled and reconstructions for gold standards fused images. (**d**) Correlation between ${\mathrm{SD}}_{21}$ and the number of nodes in reconstructions. Triangular blue points are sampled tests by randomly deleting a certain number of nodes and all downstream nodes. The line is the fitted line for those tests. The circular point and triangular red point are APP2 and our baseline. The precision (${\mathrm{SD}}_{21}$) is linearly correlated to the number of nodes reconstructed. Therefore, the relatively higher ${\mathrm{SD}}_{21}$ distances should be a byproduct of higher neurite recognition but not errors from our method

Although most of the dendritic fibers are identified, many axonal fibers are missed. Only 7% of dendritic voxels in gold standards are not identified, and the average spatial distance ${\mathrm{SD}}_{12}$ is small (4.26, Supplementary Table S3). On the other hand, the average ${\mathrm{SD}}_{12}$ and percentage of different structures (${\mathrm{PDS}}_{12}$) for axonal voxels are much higher (721.57 and 0.668, Supplementary Table S3). The reason for the poor axon recognition is there are large numbers of inter-neural fiber crossings (Fig. 6b), and UltraTracer early stops to avoid over-tracing caused by crossing.

### 3.5 Over-tracing

Over-tracing is a common problem for all tested methods, from the baseline methods, i.e. APP2 and SmartTracing, to our enhanced versions (Table 1). This is mainly caused by inter-neuronal fiber crossing. We computed self-crossing (crossing between fibers of the same neuron) as a coarse estimation of crossing. To this end, we calculated the pairwise distances between the nodes within the central (512 × 512 × 256) crop of all gold standards, after excluding nodes <50 voxels from the soma center and those sharing ancestors or offspring within 10 generations. The average number of pairs with distances <1 voxel is 3.7, showing considerable self-crossing between fibers. Assuming inter-neuronal distances share similar distribution, the inter-crossing caused over-tracing should not be uncommon.

We also conducted an interesting experiment, in which the tracing image is fused with the label image instead of segmentation. Ideally, the average ${\mathrm{SD}}_{21}$ distance should be 0. However, the average ${\mathrm{SD}}_{21}$ for APP2 tracing is 3.43. These results again highlight the existence of inter-neuronal fiber crossing and self-crossing problems under low-resolution conditions.

The problem can be further confirmed by the total path length statistics (Fig. 6c). Morphologies traced by APP2 undergo remarkable under-tracing, with very few cases greatly over-traced. After integrating with our method, there is a little bit of over-tracing, and the upper tail is supposed to be caused by the inter-neuronal crossing. As for the morphologies of the label fused images, the total length is slightly larger than the gold standards.

We also conducted additional analysis to pursue the reason why ${\mathrm{SD}}_{21}$ of our method is larger than APP2 baseline. Results show the ${\mathrm{SD}}_{21}$ values are linearly correlated with the numbers of average traced nodes (Fig. 6d), indicating that over-tracing stems from other reasons but not NeuMiner.

## 4 Discussion

In this article, we proposed a module for tracing weak fibers by introducing false-negative mining and DTGT strategies. Both strategies are demonstrated to improve the weak fibers recognition by a large margin, based on the extensive evaluation of the test set. Our study also illustrates that neuron segmentation with partial labeling can identify most fibers, which greatly relieves the annotation burden.

One limitation of NeuMiner is the use of fixed-width (3 × 3 × 1 voxels) fibers, which is inappropriate for axonal fibers, as the radii of axonal fibers are small and many of them are only 1 pixel. In this case, the (3 × 3 × 1) width ground truth may lead to artificial connections between signals, especially in the densely packed axonal arbors. The reason for choosing a fixed-width fiber is that there are small shifts between the gold standards annotated at low resolution and the high-resolution signals. We are designing strategies to remove the shifts and will then improve the segmentation accuracy of slim fibers.

Another problem for NeuMiner and all other tracing algorithms is the over-tracing caused by fibers crossing. Ideally, the over-tracing can be addressed with higher-resolution imaging systems, including optical microscopy and electric microscopy. Nevertheless, there are many difficulties for both techniques, e.g. high-resolution imaging and high-throughput data processing. An alternative solution is post-tracing pruning of over-traced neurites. There are several works on this topic (Li *et al.*, 2019a; Quan *et al.*, 2016), most of them are mainly based on angular preference, more analysis and attribution should be conducted to solve the problem.

## Supplementary Material

btac816_Supplementary_Data
