# Supplementary material for: Tracing weak neuron fibers
Source: Bioinformatics. 2022 Dec 26;39(1):btac816. doi: 10.1093/bioinformatics/btac816 (PMC9848051; doi:10.1093/bioinformatics/btac816)
Supplement: btac816_Supplementary_Data [file btac816_supplementary_data.docx]

Supplementary files

**Table S1** Ablation study for different components of NeuMiner, calibrated by Spatial Distance. FNM and DTGT are False Negative Mining and Derivative Truncated Gamma Transformation.

|  | **All neurites** | **Dendrite** | **Axon** |
| --- | --- | --- | --- |
| **APP2+NeuMiner** | 1.47/7.31/4.39 | 0.93/-/- | 1.87/-/- |
| **w/o FNM** | 2.67/6.39/4.53 | 1.15/-/- | 3.45/-/- |
| **w/o DTGT** | 1.66/6.86/4.26 | 0.93/-/- | 2.16/-/- |
| **w/o truncation** | 1.47/7.59/4.53 | 0.94/-/- | 1.86/-/- |
| **w/o segmentation** | 1.65/90.43/46.04 | 0.93/-/- | 1.91/-/- |
| **APP2** | 11.82/5.47/8.64 | 4.42/-/- | 17.86/-/- |

|  | $\boldsymbol{SD}_{\boldsymbol{12}}$**/**$\boldsymbol{SD}_{\boldsymbol{21}}$**/**$\boldsymbol{SD\downarrow}$ | | | $\boldsymbol{SSD}_{\boldsymbol{12}}$**/**$\boldsymbol{S}\boldsymbol{SD}_{\boldsymbol{21}}$**/S**$\boldsymbol{SD\downarrow}$ | $\boldsymbol{PDS}_{\boldsymbol{12}}$**/**$\boldsymbol{PDS}_{\boldsymbol{21}}$**/PDS**$\boldsymbol{\downarrow}$ | **Topology-based metrics**$\boldsymbol{\uparrow}$ | | |
| --- | --- | --- | --- | --- | --- | --- | --- | --- |
|  | **All neurites** | **Dendrite^1^** | **Axon** |  |  | $\boldsymbol{OPT-J}$ | $\boldsymbol{OPT-P}$ | $\boldsymbol{OPT-G}$ |
| **APP2** | 32.85/**3.24**/18.04 | 4.21/-/- | 50.20/-/- | 46.86/**8.92**/27.89 | 0.67/**0.13**/0.40 | 0.74 ± 0.09 | 0.58 ± 0.13 | 0.46 ± 0.17 |
| **+NeuMiner** | **9.29**/7.26/**8.27** | **1.29**/-/- | **14.36**/-/- | **25.37**/19.72/**22.54** | **0.29**/0.27/**0.28** | **0.81 ± 0.06** | **0.75 ± 0.09** | **0.67 ± 0.14** |
| **ST^2^** | 41.37/**6.36**/23.87 | 15.86/-/- | 56.60/-/- | 51.11/**11.90**/31.50 | 0.72/**0.29**/0.51 | 0.67 ± 0.20 | 0.53 ± 0.20 | 0.40 ± 0.22 |
| **+NeuMiner** | **6.28/**9.60**/7.94** | **1.15**/-/- | **9.89**/-/- | **22.20**/24.58/**23.39** | **0.21/**0.33**/0.27** | **0.78 ± 0.06** | **0.72± 0.08** | **0.62 ± 0.13** |
| ^1^ For distance metrics of separate axon or dendrite, $B_{21}$ and $B$ is meaningless as a considerable proportion of neurites are removed, so they are represented as -/-.  ^2^ 18 reconstructions are used for evaluation of SmartTracing (ST) based methods, as the others are not successfully reconstructed by SmartTracing. | | | | | | | | |

**Table S2** Quantitative comparison between base tracers and NeuMiner-enabled versions on images from new independent brains. The best values in each category are highlighted in bold. Down arrows indicate that the smaller the metrics, the better the results. Up arrows are the opposite.

**Table S3** Influence of NeuMiner on single neuron tracing

|  | **UltraTracer** | | | **UltraTracer + NeuMiner** | | |
| --- | --- | --- | --- | --- | --- | --- |
| **Metrics** | **All neurites** | **Dendrite** | **Axon** | **All neurites** | **Dendrite** | **Axon** |
| $\boldsymbol{SD}_{\boldsymbol{12}}$**/**$\boldsymbol{SD}_{\boldsymbol{21}}$**/**$\boldsymbol{SD\downarrow}$ | 853.62/**22.17**/437.89 | 5.41/-/- | 1002.80/-/- | **617.07**/55.54/**336.30** | **4.26**/-/- | **721.57**/-/- |
| $\boldsymbol{SSD}_{\boldsymbol{12}}$**/**$\boldsymbol{S}\boldsymbol{SD}_{\boldsymbol{21}}$**/S**$\boldsymbol{SD\downarrow}$ | 1031.75/**47.80**/539.78 | **17.05**/-/- | 1068.89/-/- | **841.54**/99.81/**470.67** | 19.69/-/- | **857.13**/-/- |
| $\boldsymbol{PDS}_{\boldsymbol{12}}$**/**$\boldsymbol{PDS}_{\boldsymbol{21}}$**/PDS**$\boldsymbol{\downarrow}$ | 0.729/**0.146**/0.437 | 0.161/-/- | 0.836/-/- | **0.572**/0.282/**0.427** | **0.071**/-/- | **0.668**/-/- |


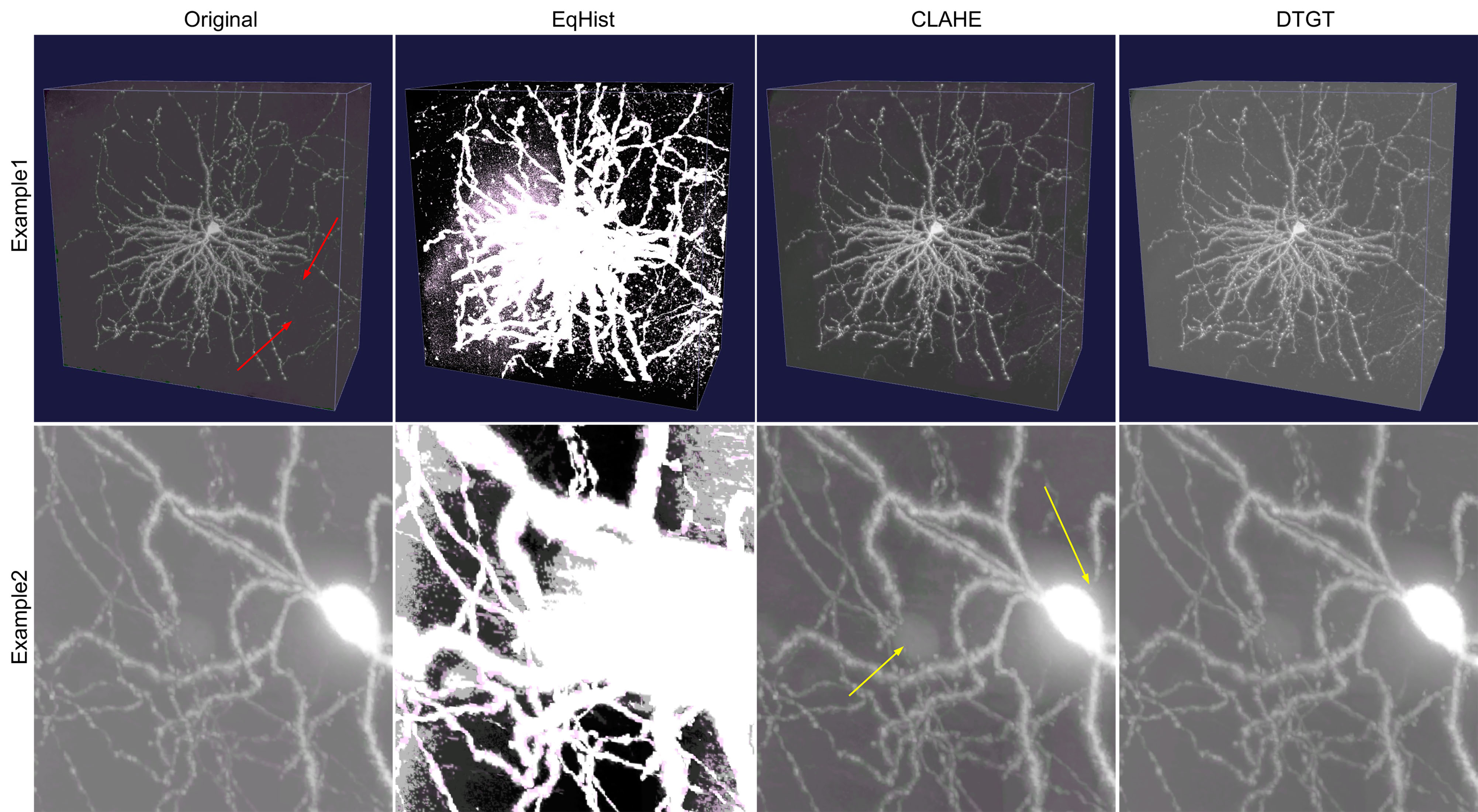


**Fig. S1**  Comparison of different image enhancers. Red arrows point out the fibers preferably enhanced by both CLAHE and DTGT. Yellow arrows illustrate the fibers suppressed by CLAHE. Global contrast normalization, e.g. histogram equalization (EqHist), leads to over-exposure for both examples. Both CLAHE and DTGT can enhance weak fibers, but CLAHE is prone to fiber suppression owing to its patch-based equalization.
